# Molecular Properties of Virulence and Antibiotic Resistance of *Pseudomonas aeruginosa* Causing Clinically Critical Infections

**DOI:** 10.3390/pathogens13100868

**Published:** 2024-10-03

**Authors:** Eric Monroy-Pérez, Jennefer Paloma Herrera-Gabriel, Elizabeth Olvera-Navarro, Lorena Ugalde-Tecillo, Luis Rey García-Cortés, Moisés Moreno-Noguez, Héctor Martínez-Gregorio, Felipe Vaca-Paniagua, Gloria Luz Paniagua-Contreras

**Affiliations:** 1 Facultad de Estudios Superiores Iztacala, Universidad Nacional Autónoma de México, Tlalnepantla 54090, Mexico; jenne9807@gmail.com (J.P.H.-G.); elizabetholverana@gmail.com (E.O.-N.); lorenau9990@gmail.com (L.U.-T.); 2 Coordinación de Investigación del Estado de México Oriente, Insitituto Mexicano del Seguro Social, Tlalnepantla de Baz 50090, Mexico; luis.garciaco@imss.gob.mx; 3 Coordinación Clínica de Educación e Investigación en Salud, Unidad de Medicina Familiar No. 55, Insitituto Mexicano del Seguro Social Estado de México Oriente, Zumpango 55600, Mexico; moises.moreno@imss.gob.mx; 4 Unidad de Biomedicina, Facultad de Estudios Superiores Iztacala, Universidad Nacional Autónoma de México, Tlalnepantla 54090, Mexico; hector.martinez@iztacala.unam.mx (H.M.-G.); felipe.vaca@iztacala.unam.mx (F.V.-P.); 5 Laboratorio Nacional en Salud, Diagnóstico Molecular y Efecto Ambiental en Enfermedades Crónico-Degenerativas, Facultad de Estudios Superiores Iztacala, Universidad Nacional Autónoma de México, Tlalnepantla 54090, Mexico

**Keywords:** *Pseudomonas aeruginosa*, multidrug resistance, virulence genes, hospital- and community-acquired infections

## Abstract

The increase in the number of hospital strains of hypervirulent and multidrug resistant (MDR) *Pseudomonas aeruginosa* is a major health problem that reduces medical treatment options and increases mortality. The molecular profiles of virulence and multidrug resistance of *P. aeruginosa*-associated hospital and community infections in Mexico have been poorly studied. In this study, we analyzed the different molecular profiles associated with the virulence genotypes related to multidrug resistance and the genotypes of multidrug efflux pumps (*mex*) in *P. aeruginosa* causing clinically critical infections isolated from Mexican patients with community- and hospital-acquired infections. Susceptibility to 12 antibiotics was determined using the Kirby–Bauer method. The identification of *P. aeruginosa* and the detection of virulence and efflux pump system genes were performed using conventional PCR. All strains isolated from patients with hospital-acquired (n = 67) and community-acquired infections (n = 57) were multidrug resistant, mainly to beta-lactams (ampicillin [96.7%], carbenicillin [98.3%], cefalotin [97.5%], and cefotaxime [87%]), quinolones (norfloxacin [78.2%]), phenicols (chloramphenicol [91.9%]), nitrofurans (nitrofurantoin [70.9%]), aminoglycosides (gentamicin [75%]), and sulfonamide/trimethoprim (96.7%). Most strains (95.5%) isolated from patients with hospital- and community-acquired infections carried the adhesion (*pilA*) and biofilm formation (*ndvB*) genes. Outer membrane proteins (*oprI* and *oprL*) were present in 100% of cases, elastases (*lasA* and *lasB*) in 100% and 98.3%, respectively, alkaline protease (*apr*) and alginate (*algD*) in 99.1% and 97.5%, respectively, and chaperone (*groEL*) and epoxide hydrolase (*cif*) in 100% and 97.5%, respectively. Overall, 99.1% of the strains isolated from patients with hospital- and community-acquired infections carried the efflux pump system genes *mexB* and *mexY*, while 98.3% of the strains carried *mexF* and *mexZ*. These findings show a wide distribution of the virulome related to the genotypic and phenotypic profiles of antibiotic resistance and the origin of the strains isolated from patients with hospital- and community-acquired infections, demonstrating that these molecular mechanisms may play an important role in high-pathogenicity infections caused by *P. aeruginosa*.

## 1. Introduction

*Pseudomonas aeruginosa* is considered one of the most important opportunistic pathogenic bacteria and causes hospital- and community-acquired infections [1], such as bacteremia, pneumonia, urinary tract infections, surgical infections, respiratory infections, and catheter-associated infections [2,3,4,5], mainly in immunocompromised patients owing to burns, HIV, cancer, catheter carriers, bone surgery, organ transplants, cystic fibrosis, and chronic obstructive pulmonary disease [6,7,8]. The pathogenicity of *P. aeruginosa* is conducted by a large number of virulence factors it possesses, among which are the adhesion gene (*pilA*) and biofilm formation gene (*ndvB*), outer membrane proteins genes (*oprL* and *oprI*), elastases genes (*lasA* and *las B*), alkaline protease gene (*apr*), alginate pseudocapsule gene (*algD*), chaperone gene (*groEL*), epoxide hydrolase gene (*cif*), and type 3 secretion system genes (T3SS) made up of four exotoxin genes (*exoT*, *exoY*, *exoS*, and *exoU*) that promote colonization, tissue destruction, and the evasion of the host immune response, increasing the acuteness, chronicity, and pathogenicity of infections [9,10,11,12,13]. The genome size of *P. aeruginosa* PAO1, which is used as a reference for comparing the genome of other strains, has been described to be around 6.3 Mbp, encoding 5700 genes, within which an estimated 150 genes encode outer membrane proteins related to adhesion, movement, antibiotics, iron uptake, and the secretion of virulence factors [14].

The treatment of hospital- and community-acquired infections caused by *P. aeruginosa* strains is a major health problem owing to the emergence of multidrug-resistant (MDR) strains [15], which decrease therapeutic options and significantly increase morbidity and mortality [16,17]. Therefore, multidrug resistant *P. aeruginosa* is recognized by the World Health Organization (WHO) as a critical priority bacterium for the research and development of new antibiotics [18]. *Pseudomonas aeruginosa* strains have different intrinsic resistance mechanisms to antibiotics, including low outer membrane permeability, the expression of efflux pumps, and the production of enzymes that inactivate antibiotics [19]. Additionally, they can acquire therapeutic resistance through the horizontal transfer of antimicrobial resistance genes that are frequently located within integrons and mobile genetic elements, such as transposons and plasmids [20]. Twelve members of the resistance-nodulation-division (RND) efflux pump family have been identified in *P. aeruginosa* [21], among which four multidrug efflux pumps (Mex) are involved in antibiotic resistance; MexAB-OprM, MexCD-OprJ, MexEF-OprN, and MexXY-OprM, facilitating the efflux of beta-lactams and quinolones, beta-lactams, quinolones, and aminoglycosides, respectively [19,22].

A high prevalence of multidrug resistance *P. aeruginosa* strains associated with hospital infections has been reported in different countries [23,24]. Notably, 28.2% of *P. aeruginosa* strains isolated in hospital-acquired infections in Mexico were found to be multidrug resistance (MDR) and 22.7% were extremely drug resistant (XDR) [25]. In the United States of America (USA), similar percentages of 20% multidrug resistance have been observed for *P. aeruginosa* strains isolated from infected children in different hospitals [26]. Among the antibiotics used to combat infections caused by *P. aeruginosa*, there are beta-lactams, aminoglycosides, quinolones, beta-lactamase inhibitors, and carbapenems [27], so the study of the resistome and the genes that code for the multidrug efflux pump systems should always be routinely analyzed in hospital strains.

This study aimed to characterize the molecular profiles linked to virulence and multidrug resistance, including the genotypes of multidrug efflux pumps (*mex*), in *P. aeruginosa* strains causing clinically critical infections. By analyzing strains isolated from Mexican patients with hospital- and community-acquired infections, the findings offer valuable insights into the molecular characteristics of *P. aeruginosa* that are reflective of those encountered in clinical settings globally.

## 2. Material and Methods

### 2.1. Origin and Identification of the Strains

From September 2022 to December 2023, a total of 124 strains of *P. aeruginosa* were collected in the Microbiology Laboratory of the Regional General Hospital No. 72 of the IMSS (Mexican Social Security Institute), a large health facility located in the municipality of Tlalnepantla de Baz, State of Mexico, Mexico. The strains were isolated from hospitalized patients with ongoing clinically relevant infections, such as bacteremia (n = 24), pneumonia (n = 16), and wound infections (n = 27), after their hospital admission for the treatment of other comorbidities, such as diabetes mellitus, chronic kidney failure, high blood pressure, and obstructive pulmonary infection. Strains were also collected from non-hospitalized patients in the external community with urinary trat infections (UTI; n = 37), respiratory infections (n = 10), and catheter-associated infections (n = 10). *Pseudomonas aeruginosa* strains were identified using VITEK 2 Compact automated equipment (bioMérieux, Marcy l’Etoile, France) and confirmed using polymerase chain reaction (PCR) analysis. All the study participants provided written informed consent. This study was approved by our institutional Ethics Committee (R-2024-1406-014).

### 2.2. Determination of Antibiotic Resistance

The Kirby–Bauer method was used to determine resistance to the following 12 antibiotics; ampicillin (AM; 10 μg), carbenicillin (CB; 100 μg), cefalotin (CF; 30 μg), cefotaxime (CFX; 30 μg), ciprofloxacin (CPF; 5 μg), norfloxacin (NOF; 10 μg), chloramphenicol (CL; 30 μg), nitrofurantoin (NF; 300 μg), amikacin (AK; 30 μg), gentamicin (GE; 10 μg), netilmicin (NET; 30 μg), and sulfamethoxazole with trimethoprim (SXT; 25 μg) (Investigación Diagnóstica, Mexico City, Mexico). The strains *P. aeruginosa* ATCC 27853 and *Escherichia coli* ATCC 25922 were used as controls for the reproducibility of the method. The criteria established by the Clinical and Laboratory Standards Institute [28] were used to interpret the results. Strains resistant to more than three antibiotics belonging to different groups were classified as multidrug resistance strains.

### 2.3. DNA Extraction

Boiling was used to extract genomic DNA from the *P. aeruginosa* strains [29]. The strains were grown on cetrimide agar (DIBICO, Edo. de México, Mexico) at 37 °C for 12 h with constant agitation. Four isolated colonies of 2 mm in diameter were collected and suspended in Eppendorf tubes with 200 μL of sterile water, incubated at 100 °C for 10 min and centrifuged at 10,000× *g* for 5 min. The DNA obtained in the supernatant was stored at −20 °C. Stock solutions of the genomic DNA of each *P. aeruginosa* strain were prepared to obtain concentrations of 100 ng/μL. DNA concentration and purity were measured using a NanoDrop 2000 spectrophotometer (Thermo Fisher Scientific, Waltham, MA, USA).

### 2.4. Identification of P. aeruginosa

*Pseudomonas aeruginosa* was identified using a PCR amplification of its 16S rDNA gene (Table S1; Supplementary Materials) [30]. For each singleplex PCR assay, a final volume of 20 µL was used per reaction mixture; 12 µL of Taq DNA Polymerase Master Mix RED (Ampliqon, Copenhagen, Denmark), 1 µL of forward primer and 1 µL of reverse primer (10 pmol, Integrated DNA Technologies, San Diego, CA, USA), 3 µL of DNA template (100 ng), and 3 µL of nuclease-free water. The following PCR amplification conditions were performed using a T100TM thermal cycler (Bio-Rad, Feldkirchen, Germany); initial denaturation at 95 °C for 2 min, 25 cycles of denaturation at 94 °C for 25 s, annealing at 58 °C for 40 s and extension at 72 °C for 40 s, and a final extension at 72 °C for 60 s. In each PCR assay, *P. aeruginosa* ATCC 27853 was used as the positive control.

### 2.5. Detection of Virulence Genes in the Strains

The primers and PCR conditions used for the identification of adhesion (*pilA*), biofilm formation (*ndvB*), outer membrane protein (*oprL* and *oprI*), elastase (*lasA* and *lasB*), alkaline protease (*apr*), alginate pseudocapsule (*algD*), chaperone (*groEL*), and epoxide hydrolase (*cif)* genes were as previously described [10,11,31,32]. The final volume per reaction mixture for each uniplex PCR assay using the T100TM thermocycler (Bio-Rad, Feldkirchen, Germany) was 20 µL; 12 μL of Taq DNA Polymerase Master Mix RED (Ampliqon), 1 μL of forward primer, 1 μL of reverse primer (10 pmol, Integrated DNA Technologies, San Diego, CA, USA), 3 μL of DNA template (100 ng), and 3 μL of nuclease-free water. In each PCR assay, *P. aeruginosa* ATCC 27853 was used as a positive control. All the primers and the genes evaluated by PCR in this study are presented in Supplementary Material (Table S1).

Group 1: The association between the frequency of virulence genotypes and the presence of genes encoding multidrug efflux pump systems was assessed in strains isolated from patients with hospital- and community-acquired infections, considering their respective diagnoses.

Group 2: The association between the antibiotic resistance phenotypes (resistome) of the strains and the corresponding patient diagnoses was evaluated.

Group 3: The association between the frequency of virulence genotypes and the presence of efflux pump system genes, in relation to the antibiotic resistance phenotypes, was examined in strains from both hospital- and community-acquired infections.

### 2.6. Unsupervised Hierarchical Clustering

The *P. aeruginosa* strains were systematically clustered according to the detection frequency of virulence genes, antibiotic resistance phenotypes, efflux pump genes, diagnosis, and clinical origin of patients with hospital- and community-acquired infections using unsupervised hierarchical clustering based on Euclidean distances for categorical variables. A categorical data matrix including virulence gene frequency, antibiotic resistance phenotype (resistance and susceptibility), efflux pump genotype, diagnosis, and clinical origin of patients with hospital- and community-acquired infections was created in R (version 3.6.2) using the *cluster* package (2.1.0). The distance between the strains was calculated based on the overall similarity coefficient, which estimated the maximum possible absolute discrepancy between each matched pair of strains. The strains were visualized in a distribution plot with a dendrogram constructed using Complex Heatmap (v3.6.2, R core).

## 3. Results

### 3.1. Virulence and Efflux Pump Genes in the Isolates

Most *P. aeruginosa* strains isolated from patients with hospital-acquired infections were from wound infections (27/67; Table 1), bacteremia (24/67), and pneumonia (16/67), whereas strains isolated from patients with community-acquired infections were derived from UTIs (37/57), respiratory infection (10/57), and catheter-associated infection (/57). The frequencies of virulence and efflux pump system genes in strains isolated from hospital- and community-acquired infections were not associated with the type of infection (*p* < 0.05; Table 1). All strains isolated from patients with hospital-acquired infections and strains isolated from patients with community-acquired infections carried outer membrane protein (*oprI* and *oprL*), elastase A (*lasA*), and chaperone (*groEL*) genes. Similarly, all strains isolated from patients with bacteremia (24/24) and strains from patients with respiratory (10/10) and catheter-associated infections (10/10) carried elastase B (*lasB*), alkaline protease (*apr*), alginate (*algD*), epoxide hydrolase (*cif*), and efflux pump system (*mexB* and *mexF*) genes. Adhesion (*pilA*) and biofilm formation (*ndvB*) genes were identified in all strains isolated in bacteremia (24/24) in patients with hospital-acquired infections and respiratory infections (10/10) in patients with community-acquired infections.

### 3.2. Resistome of Isolated Strains

The overall *P. aeruginosa* strains from patients with hospital- and community-acquired infections showed high percentages of resistance to the beta-lactam antibiotics AM (120/124), CB (122/124), CF (121/124), and CFX (108/124). Resistance to NOF (97/124), CL (114/124), NF (88/124), GE (93/124), and SXT (120/124) was also observed (Table 2). No association was found between the percentage of antibiotic resistance of the strains and the type of infection (hospital- or community-acquired) (*p* < 0.05; Table 2). Most of the strains (90–100%) from hospital-acquired infections (n = 67; bacteremia, pneumonia, and wound infection), as well as community-acquired infections strains (n = 57; UTI, respiratory infection, and catheter-associated infection), were resistant to AM, CB, CF, and SXT, while all community-acquired strains (n = 57; UTI, respiratory infection, and catheter-associated infection) were resistant to CL.

We found that 100% (n = 124; Table 3) of the strains isolated from patients with hospital-acquired (n = 67) and community-acquired (n = 57) infections were multidrug resistance to 6–12 antibiotics belonging to different groups. The highest percentages of multidrug resistance strains were observed for 12 antibiotics (43/124), followed by 8 antibiotics (24/124), 9 antibiotics (19/124), and 10 antibiotics (16/124).

### 3.3. Virulence and Efflux Pump Genotype Frequencies According to the Resistome

Overall, no statistical association was found between the frequency of virulence and efflux pump genotypes and antibiotic resistance phenotype in strains from hospital-acquired infections, except for the frequency of CL resistance, which was statistically significant (*p*-value; 0.002491, Table 4). In strains from hospital-acquired infections, high correlation percentages ranging from 89.5% (60/67) to 100% (67/67) were detected for adhesion (*pilA*), biofilm formation (*ndvB*), outer membrane protein (*oprL* and *oprI*), elastase (*lasA* and *las B*), alkaline protease (*apr*), alginate pseudocapsule (*algD*), chaperone (*groEL*), epoxide hydrolase (*cif*), and efflux pump systems (*mexB*, *mexF*, *mexY*, and *mexZ*) genes with the resistance phenotype to beta-lactams (AM, CB, CF, and CFX) and trimethoprim/sulfamethoxazole (SXT). Similarly, high correlation percentages were identified in the range of 73.1% (49/67) to 89.5% (60/67) for the virulence genotypes and efflux pumps in relation to the resistance phenotype to quinolones (NOF), phenicols (CL), and aminoglycosides (GE) (Table 4).

Regarding strains isolated from patients with community-acquired infections, no statistically significant association was found between the virulence genotype and efflux pumps in relation to the antibiotic resistance phenotype, except for CF and CL (Table 5; *p* < 0.05). Overall, the virulence genotype (*pilA*, *ndvB*, *oprL*, *oprI*, *lasA*, *lasB*, *apr*, *algD*, *groEL*, and *cif*) and efflux pumps (*mexB*, *mexF*, *mexY*, and *mexZ*) were highly correlated with beta-lactams (AM, CB, and CF), phenicols (CL), and SXT, ranging from 87.7% (50/57) to 100% (57/57). High correlation percentages of virulence genotypes and efflux pumps were also detected with beta-lactams (CFX), quinolones (NOF), nitrofurans (NF), and aminoglycosides (GE), ranging from 63.1% (36/57) to 87.7% (50/57; Table 5).

### 3.4. Genotype and Phenotype Diversity in Strains

**Figure 1:**
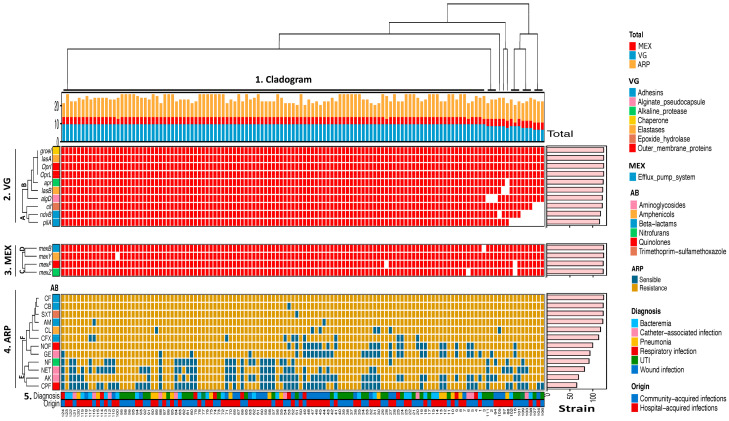
Hierarchical clustering of *P. aeruginosa* strains. The heat map is segmented into five panels, upper: (1) total cladogram of virulence genotype (VG), multidrug efflux pump (MEX), and genotype and antibiotic resistance phenotype (ARP), left: (2) VG in the upper, (3) MEX in the middle, (4) ARP in the lower, and (5) diagnosis and strain origin in the bottom panel. The right panel (pink) shows the absolute detection frequency by virulence genotype, multidrug efflux pump (MEX) and antibiotic resistance phenotype. The presence of a gene is represented in red and the absence in grey. Antibiotics (AB) are shown in Section 4. ARP (resistome). CF = Cephalothin, CB = Carbenicillin, SXT = Trimethoprim-sulfamethoxazole, AM = Ampicillin, CL = Chloramphenicol, CFX = Cefotaxime, NOF = Norfloxacin, GE = Gentamicin, NF = Nitrofurantoin, NET = Netilmicin, AK = Amikacin, and CPF = Ciprofloxacin. Antibiotic resistance is represented in yellow and susceptibility in blue.

A comprehensive analysis of the genotypic and phenotypic diversity of the strains was performed using unsupervised hierarchical clustering to better visualize their overall characteristics. Two clusters (groups 1 and 2) were identified based on the similarities between *P. aeruginosa* strains according to the virulence genotype, multidrug efflux pump system profile, antibiotic resistance phenotype, diagnosis, and strain origin (Figure 1). Group 1 comprised nine strains (102, 16, 101, 109, 103, 108, 107, 104, 106; Figure 1) distributed into three subgroups, where the frequency of the virulence genotype distribution, multidrug efflux pumps, and antibiotic resistance phenotype in strains from hospital-acquired infections (bacteremia [n = 2] and wound infection [n = 1]) and community-acquired infections (UTI [n = 5] and catheter-associated infection [n = 1]) was lower than that in Group 2. In contrast, Group 2 was larger and comprised 115 strains, distributed in five subgroups (strains 124 to 68, Figure 1), including a large subgroup with 109 strains. In general, *P. aeruginosa* strains from Group 2 patients with hospital- and community-acquired infections presented a greater amplitude of the virulence genotype related to multidrug efflux pump genes and antibiotic resistance phenotype. Analysis of the virulence genotype (VG) cladogram showed two clades (A and B), where clade B showed a higher distribution of virulence genotype composition than clade A, whereas the cladogram of the multidrug efflux pump genotype (MEX) showed a similar frequency of multidrug genes (*mex*) in both clades (C and D). Regarding the antibiotic resistance phenotype (ARP) cladogram, two clades (E and F) were detected, and clade F showed a higher prevalence of antibiotic resistance phenotypes (CF, CB, SXT, AM, CL, CFX, NOF, and GE) than clade E (NF, NET, AK, and CPF).

## 4. Discussion

Hospital- and community-acquired infections caused by multidrug resistance and hypervirulent strains of *P. aeruginosa* are major health problems that increase mortality [33,34,35,36,37]. In this study, we found a high distribution of multidrug resistance-related virulence and multidrug efflux pump (*mex*) genotypes in *P. aeruginosa* strains isolated from patients with hospital- and community-acquired infections. The adhesion (*pilA*) and biofilm formation (*ndvB*) genes were identified in all strains isolated from patients with pneumonia (16/16) and respiratory infection (10/10) with hospital-acquired infections (n = 67) and in the community (n = 57), respectively, in the same way the genes for outer membrane proteins (*oprI* and *oprL*), elastase A (*lasA*), and chaperone (*groEL*) were identified in all strains isolated from patients with bacteremia (24/24), pneumonia (16/16), and wound infection (27/27) with hospital-acquired infections (n = 67), as well as in patients with UTI (37/37), respiratory infection (10/10), and catheter-associated (10/10) community-acquired infections (n = 57), which demonstrates the high capacity of these strains to cause clinically critical infections, since it has been described that *pilA* gene is involved in adhesion and colonization [38], and *ndvB* gene promotes biofilm production, which facilitates host immune response evasion and increases antibiotic resistance [39]. *oprI* and *oprL* genes encode the main outer membrane lipoproteins involved in antibiotic resistance [40,41], *lasA* gene encodes a proteolytic enzyme that degrades host elastin [42], and GroEL stimulates the production of inflammatory cytokines, adhesion molecules, and promotes immune responses [43]. Similarly, the genes for elastase (*lasB*), alkaline protease (*apr*), alginate (*algD*), and epoxide hydrolase (*cif*) were detected in all strains associated with bacteremia (24/24) from patients with hospital infections (n = 67), as well as in respiratory infections (10/10) and catheter-associated infections (10/10) from patients with community-acquired infections (n = 57), highlighting the virulence capacity of the strains to causing severe and fatal infections in patients, especially in immunocompromised patients, because LasB activity has been described to disrupt epithelial tight junctions and degrade surfactant proteins, as well as host cytokines and immunoglobulins [44,45]. The Apr protease interferes with endothelial fibronectin and laminin, degrading cytokines and complement proteins [46], while alginate polysaccharide (AlgD) evades pulmonary phagocytosis and promotes bacterial resistance to antibiotics [47,48], and the Cif protein promotes the severity of infections in patients with cystic fibrosis and pneumonia [49]. Overall, the high percentages of the virulence genotype of more than 90% for *pilA*, *oprL*, *oprI*, *algD*, *apr*, *lasA*, *lasB*, *cif*, and *groEL* found in our strains of *P. aeruginosa* isolated from patients with hospital- and community-acquired infections were higher than those described in strains from patients with different infections in other countries; *pilA* (approximately 30%), *oprL* (29.6%), *oprI* (40.7%), *algD* (64.6% to 70.1%), *apr* (63%), *lasA* (approximately 4%), *lasB* (13% to 95.4%), and *cif* (94%) [11,31,50,51], where the frequency of *ndvB* (94.3%) and *groEL* (100%) detected in our strains was very similar *(ndvB*; 95.7% and *groEL*; 100%) to that described in strains causing chronic otitis [10].

We found a wide distribution of the efflux pump system genotype associated with hospital- and community-acquired infections, including bacteremia (24/24) and pneumonia (16/16) of patients with hospital-acquired infections (n = 67) simultaneously presenting the *mexB/mexF/mexZ* genes, while *mexB/mexF/mexY* was detected in all strains isolated from patients with respiratory (10/10) and catheter-associated (10/10) community-acquired infections (n = 57). The collective association of the *mexB*, *mexF*, *mexY*, and *mexZ* genes in hospital and community strains could be an important factor favoring the multidrug resistance phenotype because *mexY* increases resistance to aminoglycosides (tobramycin, gentamicin, and kanamycin) [52], *mexB* and *mexF* to quinolones [53,54], and *mexZ* to tobramycin [55]. In general, the high percentages of the efflux pump system genotype found in the strains show the bacterial capacity to express different profiles of the efflux pump system during infections acquired in both the hospital and community, which may reduce the available treatment options and increase hospital mortality, as demonstrated by studies of the simultaneous overexpression of the *mexY*, *mexB*, and *mexF* genes in *P. aeruginosa* strains from different infections [56,57].

In this study, all strains from hospital-acquired (n = 67) and community-acquired (n = 57) infections were found to be multidrug resistance to a range of 6–12 antibiotics, with more than 90% of the strains isolated from patients with hospital-acquired infections (n = 67; bacteremia, pneumonia, and wound infection) and community-acquired infections (n = 57; UTI, respiratory infection, and catheter-associated infection) being resistant to AM, CB, CF, and SXT. High rates of resistance were also detected for CL, CFX, NOF, NF, GE, and NET in strains associated with hospital- and community-acquired infections. The high multidrug resistance detected in these strains has been described in Mexico in *P. aeruginosa* strains (n = 92) isolated from different infections in hospitalized patients, mainly for the antibiotics trimethoprim, gatifloxacin, nitritrofurantoin, ceftazidime, amoxicillin, levofloxacin, amikacin, ampicillin, and tobramycin [25]. Overall, in our study, the resistance percentages for ciprofloxacin, kanamycin, and gentamicin, in the hospital and community strains were very similar to those found in *P. aeruginosa* strains (n = 63) isolated from skin burns in Iran [58], as well as those described in a study carried out on *P. aeruginosa* strains (n = 72) isolated from different hospital infections in Egypt, where high percentages were also detected for resistance to piperacillin/tazobactam, aztreonam, meropenem, and imipenem [33]. The molecular mechanisms developed by multidrug resistance strains include multidrug efflux pump systems [59] and the presence of extended-spectrum beta-lactamase and metallo-beta-lactamase genes, which are frequently transmitted horizontally by plasmids and transposons [60,61]. These findings suggest that the molecular determinants of *P. aeruginosa* found in this work are reflective of those encountered in clinical settings globally.

Importantly, a high correlation of more than 89% of the frequency of the adhesion (*pilA*), biofilm formation (*ndvB*), outer membrane protein (*oprI* and *oprL*), elastase (*lasA* and *lasB*), alkaline protease (*apr*), alginate (*algD*), chaperone (*groEL*), and epoxide hydrolase (*cif*) genes was found with the genotype that codes for the efflux pump systems (*mexB*, *mexF*, *mexY*, and *mexZ*), and with the resistance phenotype to beta-lactams (AM, CB, CF, and CFX), quinolones (NOF), phenicols (CL), nitrofurans (NF), and aminoglycosides (GE) in *P. aeruginosa* strains from patients with hospital-acquired infections (67/124) and also in the community (57/1245). The correlation percentages of *pilA* with the AM, CFX, CPF, and AK resistance phenotype detected in our strains were higher than those described for *P. aeruginosa* strains isolated from burn wound infections [62]. The high percentage of association of the virulence genotype with the genotype and phenotype of antibiotic resistance detected in strains from hospital and community infections represents a difficult health problem, which increases the severity of infections, decreases the options for medical treatment, and consequently, increases mortality. The virulence genotype related to the antibiotic resistance phenotype has been reported to increase the mortality rate by up to 30% (175/590) in patients with bacteremia caused by *P. aeruginosa* [63], whereas the mortality rate in other types of acute infections is 21% (4/19) [64].

Unsupervised hierarchical clustering analysis revealed a broad distribution of the virulome related to the antibiotic resistance genotype (efflux pumps), resistome, and origin of the strains isolated from hospital- and community-acquired infections. Two groups were detected, where Group 1 presented the highest number of strains (n = 115) and by very similar patterns of association of virulence genes, efflux pump systems, associated with the resistome, and the clinical origin of the infections acquired in the hospital and in the community. This led us to suppose that the hospital and community strains causing these infections are very close to each other. Whole genome sequencing of these strains may provide more detailed information on the serotypes, complete virulence determinants, and antimicrobial resistance genotypes. Our results show that complex molecular profiles of the virulence genotype and antibiotic resistance of *P. aeruginosa* cause clinically critical hospital and community infections, which may play a significant role in the their severity and therapeutic options.

## 5. Conclusions

The findings of this study provide novel insights into the complex molecular factors contributing to the genotypic and phenotypic profiles of resistance to traditionally used antibiotics and the hypervirulence associated with *P. aeruginosa* strains that cause clinically critical hospital- and community-acquired infections. The different association molecular profiles of virulence and antibiotic resistance markers found in these strains can be used to implement more effective medical treatment schemes or to generate vaccines against these important hospital pathogens.

## Figures and Tables

**Table 1:** Frequency of virulence and antibiotic resistance genes in the strains.

Function	Gene	Strain Origin (n = 124)							
		Hospital-Acquired (n = 67)			Community-Acquired (n = 57)			*p*-Value	Total No. (%)
		Bacteremia (n = 24) No. (%)	Pneumonia (n = 16) No. (%)	Wound Infection (n = 27) No. (%)	UTI (n = 37) No. (%)	Respiratory Infection (n = 10) No. (%)	Catheter-Associated Infection (n = 10) No. (%)		
Adhesins	*pilA*	22 (91.6)	16 (100)	26 (96.2)	32 (86.4)	10 (100)	9 (90)	0.5373	115 (92.7)
Biofilm formation	*ndvB*	23 (95.8)	16 (100)	25 (92.5)	34 (91.8)	10 (100)	9 (90)	0.8466	117 (94.3)
Outer membrane proteins	*oprI*	24 (100)	16 (100)	27 (100)	37 (100)	10 (100)	10 (100)	1	124 (100)
	*oprL*	24 (100)	16 (100)	27 (100)	37 (100)	10 (100)	10 (100)	1	124 (100)
Elastases	*lasA*	24 (100)	16 (100)	27 (100)	37 (100)	10 (100)	10 (100)	1	124 (100)
	*lasB*	24 (100)	15 (93.7)	26 (96.2)	37 (100)	10 (100)	10 (100)	0.5026	122 (98.3)
Alkaline protease	*apr*	24 (100)	15 (93.7)	27 (100)	37 (100)	10 (100)	10 (100)	0.5027	123 (99.1)
Alginate	*algD*	24 (100)	16 (100)	27 (100)	35 (94.5)	10 (100)	10 (100)	0.7526	121 (97.5)
Chaperone	*groEL*	24 (100)	16 (100)	27 (100)	37 (100)	10 (100)	10 (100)	1	124 (100)
Epoxide hydrolase	*cif*	24 (100)	16 (100)	26 (96.2)	35 (94.5)	10 (100)	10 (100)	0.9227	121 (97.5)
Efflux pump system (antibiotic resistance)	*mexB*	24 (100)	16 (100)	27 (100)	36 (97.2)	10 (100)	10 (100)	1	123 (99.1)
	*mexF*	24 (100)	16 (100)	27 (100)	35 (94.5)	10 (100)	10 (100)	1	122 (98.3)
	*mexY*	23 (95.8)	16 (100)	27 (100)	37 (100)	10 (100)	10 (100)	0.4839	123 (99.1)
	*mexZ*	24 (100)	16 (100)	27 (100)	36 (97.2)	10 (100)	9 (90)	0.3956	122 (98.3)

**Table 2:** Antibiotic resistance according to the origin of the strains.

Antibiotic Group	Gene	Strain Origin (n = 124)							
		Hospital-Acquired (n = 67)			Community-Acquired (n = 57)			*p*-Value	Total No. (%)
		Bacteremia (n = 24) No. (%)	Pneumonia (n = 16) No. (%)	Wound Infection (n = 27) No. (%)	UTI (n = 37) No. (%)	Respiratory Infection (n = 10) No. (%)	Catheter-Associated Infection (n = 10) No. (%)		
Beta-lactams	Ampicillin (AM)	24 (100)	16 (100)	26 (96.2)	37 (100)	10 (100)	9 (90)	0.6992	120 (96.7)
	Carbenicillin (CB)	24 (100)	16 (100)	27 (100)	37 (100)	9 (90)	10 (100)	0.1613	122 (98.3)
	Cefalotin (CF)	24 (100)	16 (100)	27 (100)	37 (100)	10 (100)	10 (100)	1	121 (97.5)
	Cefotaxime (CFX)	22 (91.6)	16 (100)	25 (92.5)	35 (94.5)	8 (80)	7 (70)	0.1026	108 (87)
Quinolones	Ciprofloxacin (CPF)	16 (66.6)	9 (56.2)	12 (44.4)	23 (62.1)	3 (30)	2 (20)	0.06182	63 (50.8)
	Norfloxacin (NOF)	21 (87.5)	13 (81.2)	20 (74)	33 (89.1)	6 (60)	7 (70)	0.217	97 (78.2)
Phenicols	Chloramphenicol (CL)	23 (95.8)	14 (87.5)	23 (85.1)	37 (100)	10 (100)	10 (100)	0.08525	114 (91.9)
Nitrofurans	Nitrofurantoin (NF)	19 (79.1)	12 (75)	18 (66.6)	31 (83.7)	7 (70)	5 (50)	0.2976	88 (70.9)
Aminoglycosides	Amikacin (AK)	17 (70.8)	9 (56.2)	14 (51.8)	23 (62.1)	3 (30)	4 (40)	0.2531	70 (56.4)
	Gentamicin (GE)	21 (87.5)	13 (81.2)	18 (66.6)	28 (75.6)	6 (60)	7 (70)	0.4363	93 (75)
	Netilmycin (NET)	17 (70.8)	12 (75)	16 (59.2)	29 (78.3)	4 (40)	4 (40)	0.08111	78 (62.9)
Sulfonamide/Trimethoprim	Sulfamethoxazole/trime thoprim (SXT)	24 (100)	16 (100)	27 (100)	36 (97.2)	10 (100)	10 (100)	1	120 (96.7)

**Table 3:** Multidrug resistance to antibiotics in strains.

Multidrug-Resistant (n = 124) (Different Antibiotic Group)	**No. of Antibiotic Groups**	**No. (%)**
	3	0 (0)
	4	0 (0)
	5	0 (0)
	6	4 (3.2)
	7	9 (7.2)
	8	24 (19.3)
	9	19 (15.3)
	10	16 (12.9)
	11	9 (7.2)
	12	43 (34.6)

**Table 4:** Distribution of virulence and antibiotic resistance genes according to the antibiotic resistance phenotype in strains isolated from patients with hospital-acquired infections.

Function	Gene	Hospital-Acquired (n = 67) No. (%)											
		Beta-Lactams				Quinolones		Phenicols	Nitrofurans	Aminoglycosides			Sulfonamide/ Trimethoprim
		AM	CB	CF	CFX	CPF	NOF	CL	NF	AK	GE	NET	SXT
Adhesins	*pilA*	63 (94)	64 (95.5)	64 (95.5)	60 (89.5)	36 (53.7)	51 (76.1)	57 (85.0)	46 (68.6)	37 (55.2)	49 (73.1)	43 (64.1)	64 (95.5)
Biofilm formation	*ndvB*	63 (94)	64 (95.5)	64 (95.5)	60 (89.5)	35 (52.2)	52 (77.6)	58 (86.5)	46 (68.6)	37 (55.2)	49 (73.1)	42 (62.6)	64 (95.5)
Outer membrane proteins	*oprI*	66 (98.5)	67 (100)	67 (100)	63 (94)	37 (55.2)	54 (80.6)	60 (89.5)	49 (73.1)	40 (59.7)	52 (77.6)	45 (67.1)	67 (100)
	*oprL*	66 (98.5)	67 (100)	67 (100)	63 (94)	37 (55.2)	54 (80.6)	60 (89.5)	49 (73.1)	40 (59.7)	52 (77.6)	45 (67.1)	67 (100)
Elastases	*lasA*	66 (98.5)	67 (100)	67 (100)	63 (94)	37 (55.2)	54 (80.6)	60 (89.5)	49 (73.1)	40 (59.7)	52 (77.6)	45 (67.1)	67 (100)
	*lasB*	64 (95.5)	65 (97)	67 (100)	61 (91.0)	36 (53.7)	52 (77.6)	58 (86.5)	48 (71.6)	38 (56.7)	50 (74.6)	45 (67.1)	65 (97)
Alkaline protease	*apr*	65 (97)	66 (98.5)	66 (98.5)	62 (92.5)	37 (55.2)	53 (79.1)	59 (88.0)	49 (73.1)	39 (58.2)	51 (76.1)	45 (67.1)	66 (98.5)
Alginate	*algD*	66 (98.5)	66 (98.5)	66 (98.5)	62 (92.5)	36 (53.7)	53 (79.1)	59 (88.0)	49 (73.1)	40 (59.7)	51 (76.1)	44 (65.6)	66 (98.5)
Chaperone	*groEL*	66 (98.5)	67 (100)	67 (100)	63 (94)	37 (55.2)	54 (80.6)	60 (89.5)	49 (73.1)	40 (59.7)	52 (77.6)	45 (67.1)	67(100)
Epoxide hydrolase	*cif*	65 (97)	66 (98.5)	66 (98.5)	62 (92.5)	37 (55.2)	53 (79.1)	59 (88.0)	48 (71.6)	39 (58.2)	51 (76.1)	44 (65.6)	66 (98.5)
Efflux pump system (antibiotic resistance)	*mexB*	66 (98.5)	67 (100)	67 (100)	63 (94)	37 (55.2)	54 (80.6)	60 (89.5)	49 (73.1)	40 (59.7)	52 (77.6)	45 (67.1)	67 (100)
	*mexF*	66 (98.5)	67 (100)	67 (100)	63 (94)	37 (55.2)	54 (80.6)	60 (89.5)	49 (73.1)	40 (59.7)	52 (77.6)	45 (67.1)	67 (100)
	*mexY*	65 (97)	66 (98.5)	66 (98.5)	62 (92.5)	37 (55.2)	54 (80.6)	49 (73.1)	48 (71.6)	39 (58.2)	51 (76.1)	44 (65.6)	66 (98.5)
	*mexZ*	66 (98.5)	67 (100)	67 (100)	63 (94)	37 (55.2)	54 (80.6)	60 (89.5)	49 (73.1)	40 (59.7)	52 (77.6)	45 (67.1)	67 (100)
*p*-value		0.8315	0.1377	0.1377	0.9972	1	1	**0.002491**	1	1	1	1	0.1161

Significant *p*-values (<0.05) are shown in bold. AM = Ampicillin; CB = Carbenicillin; CF = Cefalotin; CFX = Cefotaxime; CPF = Ciprofloxacin; NOF = Norfloxacin; CL = Chloramphenicol; NF = Nitrofurantoin; AK = Amikacin; GE = Gentamicin; NET = Netilmycin; SXT = Sulfamethoxazole/trimethoprim.

**Table 5:** Distribution of virulence and antibiotic resistance genes according to the antibiotic resistance phenotype in strains isolated from patients with community-acquired infections.

Function	Gene	Community-Acquired Infections (n = 57)											
		Beta-lactams				Quinolones		Phenicols	Nitrofurans	Aminoglycosides			Sulfonamide/Trimethoprim
		AM	CB	CF	CFX	CPF	NOF	CL	NF	AK	GE	NET	SXT
Adhesins	*pilA*	50 (87.7)	50 (87.7)	51 (89.4)	44 (77.1)	26 (45.6)	41 (71.9)	51 (89.4)	38 (66.6)	26 (45.6)	36 (63.1)	34 (59.6)	50 (87.7)
Biofilm formation	*ndvB*	52 (91.2)	52 (91.2)	53 (92.9)	46 (80.7)	27 (47.3)	42 (73.6)	53 (92.9)	40 (70.1)	26 (45.6)	37 (64.9)	35 (61.4)	52 (91.2)
Outer membrane proteins	*oprI*	56 (98.2)	56 (98.2)	57 (100)	50 (87.7)	28 (49.1)	46 (80.7)	57 (100)	43 (75.4)	30 (52.6)	41 (71.9)	37 (64.9)	56 (98.2)
	*oprL*	56 (98.2)	56 (98.2)	57 (100)	50 (87.7)	28 (49.1)	46 (80.7)	57 (100)	43 (75.4)	30 (52.6)	41 (71.9)	37 (64.9)	56 (98.2)
Elastases	*lasA*	56 (98.2)	56 (98.2)	57 (100)	50 (87.7)	28 (49.1)	46 (80.7)	57 (100)	43 (75.4)	30 (52.6)	41 (71.9)	37 (64.9)	56 (98.2)
	*lasB*	56 (98.2)	56 (98.2)	57 (100)	50 (87.7)	28 (49.1)	46 (80.7)	57 (100)	43 (75.4)	31 (54.3)	41 (71.9)	37 (64.9)	56 (98.2)
Alkaline protease	*apr*	56 (98.2)	56 (98.2)	57 (100)	50 (87.7)	28 (49.1)	46 (80.7)	57 (100)	43 (75.4)	30 (52.6)	41 (71.9)	37 (64.9)	56 (98.2)
Alginate	*algD*	54 (94.7)	54 (94.7)	55 (96.4)	48 (84.2)	27 (47.3)	44 (77.1)	55 (96.4)	42 (73.6)	30 (52.6)	40 (70.1)	36 (63.1)	54 (94.7)
Chaperone	*groEL*	56 (98.2)	56 (98.2)	57 (100)	50 (87.7)	28 (49.1)	46 (80.7)	57 (100)	43 (75.4)	30 (52.6)	41 (71.9)	37 (64.9)	56 (98.2)
Epoxide hydrolase	*cif*	54 (94.7)	54 (94.7)	55 (96.4)	48 (84.2)	27 (47.3)	44 (77.1)	55 (96.4)	41 (71.9)	28 (49.1)	39 (68.4)	35 (61.4)	54 (94.7)
Efflux pump system (antibiotic resistance)	*mexB*	55 (96.4)	55 (96.4)	56 (98.2)	49 (85.9)	27 (47.3)	45 (78.9)	56 (98.2)	42 (73.6)	29 (50.8)	40 (70.1)	36 (63.1)	55 (96.4)
	*mexF*	54 (94.7)	54 (94.7)	55 (96.4)	48 (84.2)	26 (45.6)	45 (78.9)	55 (96.4)	41 (71.9)	29 (50.8)	40 (70.1)	35 (61.4)	54 (94.7)
	*mexY*	56 (98.2)	56 (98.2)	57 (100)	50 (87.7)	28 (49.1)	45 (78.9)	57 (100)	43 (75.4)	30 (52.6)	41 (71.9)	37 (64.9)	56 (98.2)
	*mexZ*	54 (94.7)	54 (94.7)	55 (96.4)	48 (84.2)	27 (47.3)	46 (80.7)	55 (96.4)	41 (71.9)	30 (52.6)	41 (71.9)	35 (61.4)	54 (94.7)
*p*-value		0.2267	0.2267	**0.001567**	0.9592	1	0.9889	**0.001567**	0.9989	1	0.9987	0.9954	0.2267

Significant *p*-values (<0.05) are shown in bold. AM = Ampicillin; CB = Carbenicillin; CF = Cefalotin; CFX = Cefotaxime; CPF = Ciprofloxacin; NOF = Norfloxacin; CL = Chloramphenicol; NF = Nitrofurantoin; AK = Amikacin; GE = Gentamicin; NET = Netilmycin; SXT = Sulfamethoxazole/trimethoprim.

## Data Availability

The original contributions presented in the study are included in the article/Supplementary Materials.

## Supplementary Materials

The following supporting information can be downloaded at: https://www.mdpi.com/article/10.3390/pathogens13100868/s1, Table S1: List of primers used in this study.
