# Supplementary material for: Molecular Properties of Virulence and Antibiotic Resistance of Pseudomonas aeruginosa Causing Clinically Critical Infections
Source: Pathogens. 2024 Oct 3;13(10):868. doi: 10.3390/pathogens13100868 (PMC11510431; doi:10.3390/pathogens13100868)
Supplement: Supplementary file 1 [file pathogens-13-00868-s001.zip › pathogens-3220342-supplementary.pdf]

Table S1. List of primers used in this study.

Gene	Direction	Primer sequences (5′-3′)	Product size (pb)
16SrDNA	Forward<br>Reverse	GGGGGATCTTCGGACCTCA<br>TCCTTAGAGTGCCCACCCG	956
		**Virulence gene**	
pilA	Forward<br>Reverse	AGCTCAAAAAGGCTTTACCTTGAT<br>ACTTGGTCAGCGACATACGTAATA	315
ndvB	Forward<br>Reverse	GAAGGCTACAGCTACTTCGTCATC<br>CTGTACTGCTGGCTGTAGTCGTAG	317
oprL	Forward<br>Reverse	ATGGAAATGCTGAAATTCGGC<br>CTTCTTCAGCTCGACGCGACG	504
oprI	Forward<br>Reverse	ATGAACAACGTTCTGAAATTCTCTGCT<br>CTTGCGGCTGGCTTTTTCCAG	249
apr	Forward<br>Reverse	ATCCTGGTACCTGATCAACAGC<br>TGTAGCTCATCACCGAATAGGC	203
algD	Forward<br>Reverse	CGTCTGCCGCGAGATCGGCT<br>GACCTCGACGGTCTTGCGGA	313
groEL	Forward<br>Reverse	AGCCATGGAAAAAGTCGGTAAAG<br>CGG ATGTTGGAGATCTTCTTGT	344
cif	Forward<br>Reverse	TGATGCTGGTACACGGTTTC<br>CGTCCGCATAGGCTTTCAT	687
		**efflux pump gene**	
mexB	Forward<br>Reverse	CCTGCTGATCTACGTGGTGA<br>CCTTCTCCAGCAGGTATTCG	182
mexF	Forward<br>Reverse	TCTACGACCCGACCATCTTC<br>AGGAACAGGATCACCACCAG	100
mexY	Forward<br>Reverse	CAACGGCTATCCCTCGTTCA<br>AACACGATCAGCACCGAGAG	198
mexZ	Forward<br>Reverse	TGGCCAGAAAAACCAAAGAG<br>CAGGCAGACCTCGATCTTGT	179
